# Pre-Analytical and Analytical Challenges in Whole-Exome Sequencing of Formalin-Fixed Paraffin-Embedded Breast and Prostate Cancer Tissue: A Real-World Multicenter Study

**DOI:** 10.3390/diagnostics16111595

**Published:** 2026-05-23

**Authors:** Mahira L. Rosa, Cláudia Bordignon, Jaqueline B. Schuch, Angélica C. Baumont, Marina Bessel, Giovana D. Curzel, Nathan A. Cadore, Ana Paula M. Varela, Giovana T. dos Santos, Tiago F. Andreis, Francine H. Oliveira, Vitor F. Vasconcellos, Lilian A. R. Barros, Cristiano P. Souza, Williams F. Barra, Daniela L. C. Louzeiro, Alessandra Notari, Juliana J. de Menezes, Pedro E. R. Liedke, Gláucio A. Bertollo, Aline B. L. Gongora, Henrique G. Ascenco, Eduardo Kowalski-Neto, Christina P. Oppermann, Gustavo Werutsky, Edilmar M. Santos, Flavio S. Brandão, Ruffo Freitas, Jr., Angélica Nogueira-Rodrigues, André L. C. Mancini, Daniela D. Rosa, Gabriel S. Macedo

**Affiliations:** 1 Hospital Moinhos de Vento, Porto Alegre 90540-000, RS, Brazil; mahira.rosa@hmv.org.br (M.L.R.); marina.bessel@hmv.org.br (M.B.);; 2 Hospital Universitário Cassiano Antônio Moraes, Vitória 29040-090, ES, Brazil; 3 Instituto Brasileiro de Controle do Câncer, São Paulo 03164-000, SP, Brazil; 4 Hospital de Câncer de Barretos, Barretos 14784-400, SP, Brazil; 5 Núcleo de Pesquisas em Oncologia, Universidade Federal do Pará, Belém 66073-005, PA, Brazil; 6 Hospital de Oncologia Dr. Tarquinio Lopes Filho, São Luís 65076-090, MA, Brazil; 7 Hospital Escola da Universidade Federal de Pelotas, Empresa Brasileira de Serviços Hospitalares (EBSERH), Pelotas 96020-360, RS, Brazil; 8 Hospital Nossa Senhora da Conceição, Porto Alegre 91350-200, RS, Brazil; 9 Hospital de Clínicas de Porto Alegre, Porto Alegre 90035-903, RS, Brazil; 10 Associação Feminina de Educação e Combate ao Câncer (AFECC), Hospital Santa Rita de Cássia, Vitória 29052-010, ES, Brazil; 11 Hospital do Câncer UOPECCAN, Cascavel 85807-200, PR, Brazil; 12 Hospital Universitário Maria Aparecida Pedrossian, Campo Grande 79080-190, MS, Brazil; 13 Hospital Calixto Midlej Filho, Santa Casa de Misericórdia de Itabuna, Itabuna 45603-050, BA, Brazil; 14 Faculty of Medicine, Universidade Estadual de Santa Cruz (UESC), Ilhéus 45662-900, BA, Brazil; 15 Hospital Fêmina, Porto Alegre 90540-000, RS, Brazil; 16 Hospital São Lucas, Pontifícia Universidade Católica do Rio Grande do Sul (PUCRS), Porto Alegre 90610-000, RS, Brazil; 17 Liga Norte Riograndense Contra o Câncer, Natal 59075-740, RN, Brazil; 18 Santa Casa de Belo Horizonte, Belo Horizonte 30150-221, MG, Brazil; 19 Hospital Araújo Jorge, Associação de Combate ao Câncer em Goiás, Goiânia 74605-050, GO, Brazil; 20 Faculty of Medicine, Universidade Federal de Minas Gerais, Belo Horizonte 31270-901, MG, Brazil; 21 Hospital Universitário Getúlio Vargas, Manaus 69020-170, AM, Brazil; 22 Instituto D’Or de Pesquisa e Ensino (IDOR), São Paulo 04538-132, SP, Brazil

**Keywords:** breast neoplasms, exome sequencing, precision medicine, prostatic neoplasms

## Abstract

**Background/Objectives**: Formalin-fixed paraffin-embedded tissue is widely used in pathology and molecular diagnostics, yet its variable quality can critically influence the accuracy of sequencing-based analyses. This study investigated pre-analytical and analytical factors that can affect exome sequencing performance in a Brazilian multicenter cohort of patients with breast cancer and prostate adenocarcinoma. **Methods**: Tumor samples were reviewed for diagnostic confirmation, and those with a minimum cellularity of 20% underwent DNA extraction, fluorometric quantification, fragmentation analysis, and exome sequencing. Pre-analytical parameters, including tumor content, DNA yield, and fragmentation profile, were recorded and correlated with sequencing results. **Results**: Only 36.7% of all analyzed samples (163/444) generated valid whole-exome sequencing data, corresponding to 55.6% of those that proceeded to sequencing (163/293). Although 94.5% of specimens met the minimum ≥20% cellularity threshold and 66.0% advanced to sequencing, a substantial proportion failed to yield usable exome data. Successful sequencing was associated with shorter storage durations (*p* < 0.001) and superior analytical parameters (higher autosomal coverage, longer read lengths, lower duplication rates, and higher target coverage at ≥500× and ≥100×; *p* < 0.001). Detailed fixation-related variables (e.g., formalin type, fixation time, ischemia time) were not consistently available across centers, representing a major limitation for causal interpretation of pre-analytical effects. **Conclusions**: Our study identified a high failure rate in sequencing archival tissue, highlighting the need to prioritize more recently collected specimens and refine standardized sample handling protocols to maintain DNA integrity. These improvements are essential for optimizing sequencing workflow performance and feasibility in real-world settings.

## 1. Introduction

Precision medicine in cancer care has evolved rapidly and become standard practice in recent years, enabling the identification of multiple actionable genomic alterations in tumors. Advances in precision oncology aim to tailor diagnosis and treatment to the specific molecular characteristics of an individual’s tumor, thereby improving patient outcomes [1]. A key aspect of this approach is the identification of somatic driver mutations—genetic alterations that accumulate over time and promote cancer progression through mechanisms such as proliferation, invasion, and metastasis [2]. The need for this understanding has led to an increase in clinical trials, enabling the incorporation of several targeted therapies into cancer treatment [3].

Next-generation sequencing (NGS) technologies have been widely employed in the field of precision medicine to detect clinically relevant somatic mutations across various cancer subtypes [4]. In recent years, comprehensive panels containing hundreds of genes have become routinely used in the somatic setting for tumor profiling. This trend toward increasingly broader genomic assessments, including whole-exome sequencing (WES), has been driven, in part, by the growing clinical relevance of genomic signatures such as tumor mutational burden, microsatellite instability, and homologous recombination deficiency-associated mutational signatures, which have gained significant importance as biomarkers for therapeutic decision-making and tumor classification [5,6]. The WES provides comprehensive coding-region coverage beyond predefined actionable hotspots, supporting exploratory analyses such as mutational signatures and broader biomarker discovery. Importantly, exome-wide profiling requires consistent library complexity and relatively uniform coverage across a large capture territory; therefore, WES performance is often more affected by FFPE-associated DNA fragmentation and chemical damage than smaller targeted panels. In this context, WES offers a stringent approach to assess the impact of real-world pre-analytical variability in archival tumor specimens [5,7].

NGS has been applied to breast cancer (BC) and prostate cancer (PC), two of the most prevalent malignancies worldwide [8]. Several studies have demonstrated the impact of molecular testing on clinical outcomes in both tumor types [9,10,11,12]. In a real-world setting, comprehensive genomic profiling (CGP) in routine clinical practice identified potentially actionable genomic alterations in 57% of PC cases and 38% of HER2-positive BC cases [13,14]. However, although the molecular landscapes of BC and PC have been extensively characterized in Europe, the United States, and parts of Asia, data from Latin American populations, including Brazil, remain limited [15,16].

As clinical guidelines continue to incorporate a growing number of actionable genes, CGP is becoming increasingly essential in cancer management [17]. In this context, formalin-fixed paraffin-embedded (FFPE) tumor is a well-established and widely used source of biological material for molecular testing, as it allows long-term preservation of tumor specimens at room temperature while maintaining tissue architecture and tumor-specific information. Although FFPE samples represent a powerful tool in cancer genome analysis, several factors can affect sequencing success and, consequently, limit the identification of somatic genomic alterations [18,19]. Formaldehyde can affect the DNA double helix by interacting with different DNA structures through the formation of crosslinks, compromising the quality of DNA extracted from FFPE samples [20]. Insufficient DNA input can contribute to failed library preparation as well as negatively affect sequencing metrics and genomic variant detection [21,22,23,24]. These FFPE-associated molecular alterations can directly translate into downstream sequencing failure, including unsuccessful library preparation, increased duplication rates, and insufficient sequencing coverage, ultimately resulting in inconclusive or unusable WES data [25].

Although the determinants of WES success in FFPE tissue are known, real-world evidence quantifying sequencing attrition in multicenter healthcare systems remains scarce, particularly in Latin America. Therefore, using FFPE tumor specimens from a Brazilian cohort of BC and PC patients, we performed an exploratory analysis to investigate the impact of pre-analytical and analytical variables on WES performance and quality metrics. This study provides a pragmatic estimate of sequencing yield in routine archival samples and highlights factors correlated with WES feasibility under heterogeneous real-world conditions.

## 2. Materials and Methods

### 2.1. Study Design and Eligibility

This study is part of the Onco-Genomas Brasil project (NCT05306600), a multicenter observational cohort study designed to evaluate somatic and germline molecular alterations in patients with early-stage HER2-positive BC (arm 1, *n* = 252) and metastatic PC (arm 2, *n* = 192) treated within the Brazilian public health care system [26]. Participants were recruited from 19 hospitals across all 5 geographic regions of Brazil. Arm 1 included women aged ≥18 years with stage II or III HER2-positive BC undergoing neoadjuvant chemotherapy combined with anti-HER2 therapy (including at least trastuzumab). Arm 2 included men aged ≥ 18 years with metastatic prostate adenocarcinoma. The main exclusion criterion for both groups was the unavailability of biological samples, specifically blood and FFPE tumor samples. For all participants, clinical and pathological data were collected along with the corresponding biological specimens.

The Onco-Genomas Brasil study is part of the Brazilian Genomes Program (Programa Genomas Brasil), which is supported by the Brazilian Ministry of Health. The study protocol was previously published by Schuch et al. [26].

### 2.2. Tumor Samples—Collection, Diagnostic Confirmation, DNA Extraction, and Sequencing

FFPE tumor tissue and blood samples were requested for somatic and germline WES, respectively. Tumor specimens were defined as those previously collected during routine diagnostic or clinical care procedures. In arm 1, BC samples were obtained from diagnostic biopsies, performed prior to the initiation of systemic therapy. In arm 2, PC samples were obtained from either the primary tumor or relapsed disease, provided they originated from soft tissue (bone tissue samples were excluded). All tumor samples were transported to the coordinating center for diagnostic confirmation by a specialized local pathologist. Samples with a confirmed diagnosis and a minimum tumor content of 20% were subsequently reviewed by a pathologist, who delineated the tumor area on histological slides before DNA extraction.

Genomic DNA (gDNA) was extracted from FFPE tissue samples using 10-µm sections per sample and the QIAamp DNA FFPE Tissue kit (Qiagen, Redwood City, CA, USA). Tumor gDNA quantification was performed with the Qubit™ 1X dsDNA High Sensitivity kit (Invitrogen™, Carlsbad, CA, USA) on the Qubit™ 4.0 fluorometer (Invitrogen™, Carlsbad, CA, USA), followed by fragmentation analysis on the Agilent 4150 TapeStation system (©Agilent Technologies, Santa Clara, CA, USA) using the Agilent D5000 ScreenTape System kit (©Agilent Technologies, Santa Clara, CA, USA). All extracted DNA samples were stored at −20 °C until sequencing. Samples yielding at least 100 ng (2.5 ng/µL) of gDNA were subjected to WES on the Illumina NovaSeq6000 platform (Illumina, San Diego, CA, USA). The workflow included library preparation with the Twist Library Preparation EF 2.0 Kit(Twist Bioscience, South San Francisco, CA, USA), target enrichment using the Twist Target Enrichment Standard Hybridization v2 Kit (Twist Bioscience, South San Francisco, CA, USA), and sequencing with the NovaSeq 6000 S4 Reagent Kit (Illumina, San Diego, CA, USA). Paired normal-tumor sequence reads were mapped and aligned to the human reference genome hg38 using the Illumina^®^ DRAGEN (Dynamic Read Analysis for GENomics) pipeline, version 3.10.4 (Illumina, San Diego, CA, USA).

Samples were considered to generate valid WES data when they achieved a minimum average autosomal target coverage of ≥200×, and coverage uniformity of ≥95% across the targeted regions. NGS outcomes were classified as: failed library preparation, defined as the inability to generate sequencing libraries meeting minimal quality control requirements for sequencing; inconclusive WES, corresponding to samples with insufficient sequencing depth and coverage uniformity to support reliable downstream variant calling and clinical-grade interpretation; and valid WES data, defined as those achieving adequate coverage completeness and duplication thresholds for clinical-grade interpretation.

Importantly, this study was not designed to evaluate variant-level performance, including accuracy, concordance with matched samples, or clinical interpretability of detected alterations. The analytical framework focused exclusively on sequencing feasibility, workflow performance, and quality control metrics.

### 2.3. Statistical Analysis

Statistical analyses were performed using R version 4.2.2 (R Foundation for Statistical Computing, Vienna, Austria). A two-sided *p*-value < 0.05 was considered statistically significant.

The analytical strategy aimed to evaluate the association between pre-analytical variables (sample age, tumor cellularity, DNA yield, and fragmentation profile) and WES success, as well as sequencing quality control metrics. Sample age was defined as the time elapsed between FFPE block preparation and DNA extraction.

Categorical variables included WES outcome status (failed, inconclusive, valid) and cancer type (BC and PC), and were expressed as absolute frequencies (*n*) and percentages (%). Continuous variables (sample age, tumor cellularity, DNA concentration, fragment length, and coverage metrics) were expressed as means and standard deviations (SD).

Spearman correlation coefficients were calculated to assess the relationship between sample age, DNA mass, and DNA fragmentation, as well as between average autosomal coverage of target regions and sample age, tumor cellularity, DNA mass, and DNA fragmentation.

Associations between WES status (failed library preparation, inconclusive, or conclusive results) and sample age, tumor cellularity, DNA mass, DNA fragmentation, sequencing coverage, and read length were evaluated using the Mann–Whitney U test or Kruskal–Wallis test, as appropriate. Bonferroni correction was applied for pairwise comparisons.

To identify independent predictors of sequencing success, a multivariable Poisson regression model with robust variance and log link was fitted. Given the outcome prevalence, this approach was preferred over logistic regression to directly estimate adjusted prevalence ratios (PRs). Continuous predictors were rescaled to clinically meaningful increments, and skewed variables were log-transformed prior to inclusion in the model.

## 3. Results

A total of 444 FFPE tumor samples were assessed. The mean sample age was 27.9 months, with an average tumor cellularity of 59.8%. Only 5.5% (*n* = 24) of the samples exhibited cellularity below the 20% threshold. Four eligible patients had insufficient tumor tissue for further analysis. DNA was then extracted from the remaining 416 samples, 107 of which (25.7%) yielded less than 100 ng. PC samples were significantly older (37.6 vs. 20.6 months; *p* < 0.001), had higher tumor cellularity (73.9% vs. 49.0%; *p* < 0.001), and had lower DNA concentrations (11.94 vs. 18.22 ng/μL; *p* < 0.001) than BC samples. The average DNA fragment length was 1105 bp (mean peak = 658 bp), with similar values between PC and BC samples (Table 1).

**Figure 1:**
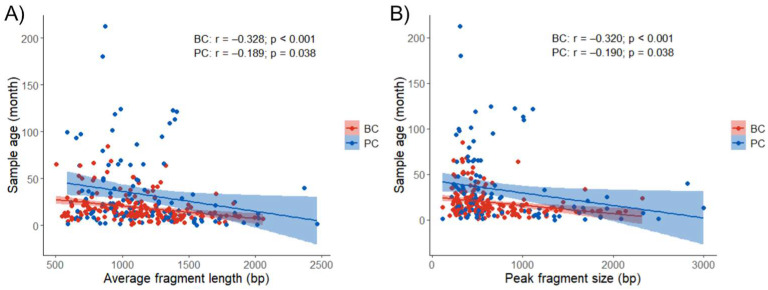
Correlations between sample age and DNA integrity in BC and PC samples. (**A**) Spearman correlation between sample age and average fragment length. (**B**) Spearman correlation between sample age and peak fragment size. Each point represents an individual sample (red for BC and blue for PC). The solid line indicates the fitted correlation, with the shaded area representing the confidence interval. Spearman’s correlation coefficient and the corresponding *p*-value are shown for each analysis.

In BC samples, sample age was inversely correlated with average fragment length (r = −0.328; *p* < 0.001) and peak fragment size (r = −0.320; *p* < 0.001), but not with DNA concentration (r = −0.086; *p* = 0.192). In PC samples, older samples showed a significant negative correlation with DNA integrity (r = −0.189 for average fragment length; r = −0.190 for peak fragment size; *p* = 0.038 for both) and DNA quantification (r = −0.152; *p* = 0.039). These findings suggest that prolonged storage harms DNA integrity (Figure 1).

**Figure 2:**
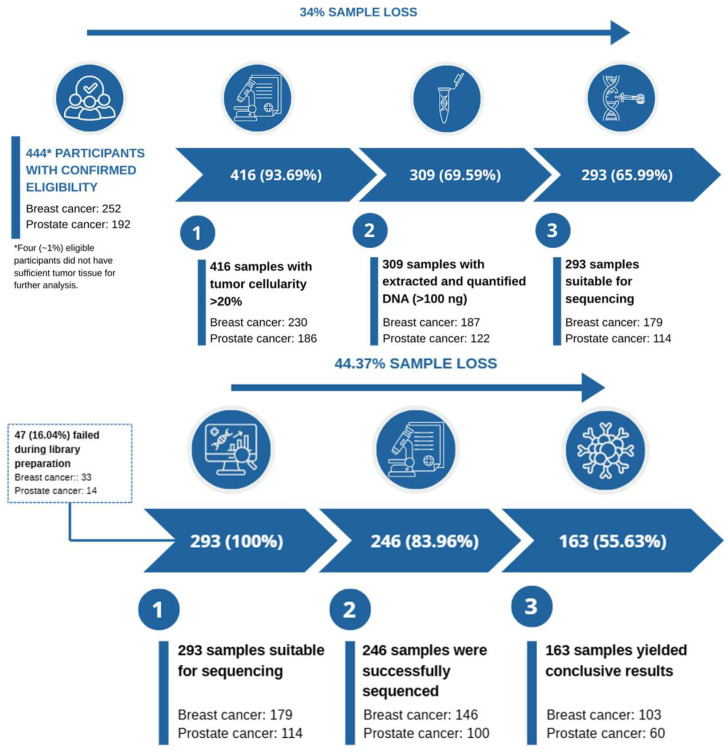
Flowchart of analytical steps and the number of samples at each stage of processing.

Although 309 samples achieved the minimum DNA input threshold, 16 were not submitted for sequencing due to poor DNA fragmentation profiles. WES was performed on 293 tumor samples, comprising 179 BC and 114 PC samples. In addition to the initial 34% pre-analytical loss, sequencing failed in 44.3% of samples. Among the 179 BC samples, 33 failed during library preparation and 43 did not meet the minimum coverage quality control metrics, with 103 samples yielding sequencing data. Among the 114 PC samples, 14 failed during library preparation and 40 did not meet the minimum coverage quality control metrics, with only 60 samples yielding sequencing data. Therefore, among all tumor samples subjected to WES, only 55.6% (*n* = 163/293) generated valid sequencing data (Figure 2). When considering the entire cohort initially assessed (*n* = 444), the overall success rate was 36.7%. Samples were classified as valid when sequencing achieved sufficient depth and completeness for downstream variant calling, whereas inconclusive samples corresponded to those with substantially reduced effective coverage and poor metrics, limiting analytical reliability. No significant differences were observed in key WES quality metrics between BC and PC samples (Table 2).

**Figure 3:**
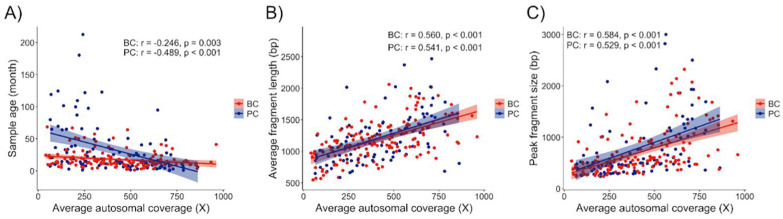
Correlations between autosomal coverage and pre-analytical parameters in BC and PC samples. Spearman correlation between average autosomal coverage and sample age (**A**), average fragment length (**B**), and peak fragment size (**C**). Each point represents an individual sample (red for BC and blue for PC). The solid line indicates the fitted correlation, with the shaded area representing the confidence interval. Spearman’s correlation coefficient and the corresponding *p*-value are shown for each analysis.

Average autosomal coverage was correlated with sample age and fragment length in both tumor types. Sample age was negatively correlated with average autosomal coverage (Figure 3A), whereas longer fragment length (average and peak) was correlated with higher average autosomal coverage (Figure 3B,C). Tumor cellularity and DNA concentration were not significantly correlated with average autosomal coverage (respectively, BC: r = −0.058, *p* = 0.484; r = 0.054, *p* = 0.514; and PC: r = 0.060, *p* = 0.0553; r = −0.097, *p* = 0.337; Figure S1).

Samples that generated valid NGS data had shorter storage durations, similar DNA amounts, and longer fragment lengths than those with inconclusive results. Overall, samples with failed library preparation had worse pre-analytical quality metrics (Table 3). Compared with samples with inconclusive results, those with valid NGS data exhibited substantially higher autosomal coverage, longer read lengths, lower duplication rates, and a higher proportion of target regions covered at ≥500× and ≥100× (Table 4).

**Figure 4:**
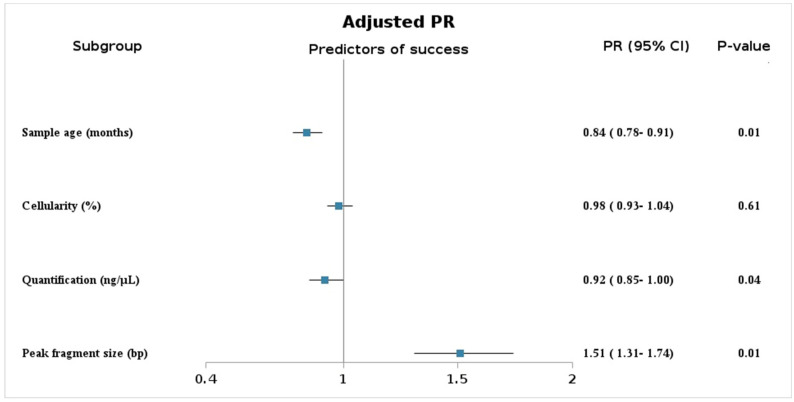
Forest plot for independent predictors of WES success.

In the multivariable Poisson regression model (Figure 4; Table S1) with robust variance, increasing sample age (per 1-year increment) was independently associated with lower sequencing success (adjusted PR = 0.84, 95% CI: 0.78–0.91; *p* = 0.01). Tumor cellularity, modeled per 15% increase, was not independently associated with sequencing outcome (adjusted PR = 0.98, 95% CI: 0.93–1.04; *p* = 0.61). Log-transformed DNA quantification was modestly associated with sequencing success (adjusted PR = 0.92, 95% CI: 0.85–1.00; *p* = 0.04). Log-transformed peak fragment size was strongly associated with higher sequencing success (adjusted PR = 1.51, 95% CI: 1.31–1.74; *p* = 0.01).

## 4. Discussion

FFPE tumor samples from breast and prostate tissue biopsies served as the source of genetic material for evaluating genomic alterations and signatures. The present study identified a high proportion of samples unsuitable for sequencing, underscoring a critical barrier to successful genomic profiling using archival tissue. Approximately one-third of all collected samples were excluded during the pre-analytical phase due to insufficient tumor cellularity or DNA quantity and quality. After accounting for additional losses due to analytical failures, such as failed library preparation and poor sequencing quality—primarily driven by insufficient average target coverage and/or low coverage uniformity across target regions—only 36.7% of the total assessed sample yielded valid sequencing data. Thus, valid WES data correspond to 55.6% of sequenced samples. This overall success rate is considerably lower than those reported in comparable studies, ranging from 58% to 82% [27,28,29,30,31]. These studies predominantly employed targeted NGS panels with smaller capture sizes, larger DNA input and more controlled experimental conditions. Additionally, many of these analyses were conducted in optimized laboratory settings or using prospectively collected or more recently processed specimens, which may not reflect the variability encountered in real-world multicenter cohorts such as ours. Although methodological differences across studies—such as sequencing platforms, DNA input requirements, capture design, and target depth—limit direct comparison, our results still indicate a persistently high failure rate of archival FFPE samples.

NGS is the method of choice for detecting clinically relevant genomic alterations in tumor samples [7,32]. More comprehensive approaches, such as CGP and WES, impose stricter quality metrics and require higher DNA integrity, compared with target panels. Although fresh-frozen samples are the gold standard to evaluate genomic alterations, clinical whole-genome sequencing has proven feasible from routine FFPE samples [6], and large-scale analyses confirm the preservation of substantial clinical utility. FFPE-derived DNA is particularly susceptible to artifacts such as cytosine deamination, which may increase false-positive variant calls if not adequately controlled. In this context, bioinformatic filtering and stringent quality thresholds play a critical role in mitigating these effects and preserving analytical reliability [7]. Multiple studies have demonstrated that, when adequate quality-control thresholds are applied, including DNA quality, sequencing depth, and bioinformatic processing, FFPE-derived WES can achieve high concordance with matched fresh-frozen samples [6,23,33,34]. However, reduced concordance has been reported when DNA integrity is compromised or when low-frequency variants are analyzed without stringent filtering criteria [35], highlighting the distinction between technical feasibility and analytical validity. To maintain confidence in variant detection, we adopted conservative analytical thresholds in our workflow, acknowledging that this strategy may reduce the overall number of analyzable samples. The absence of a comparator arm—including fresh-frozen tissue, prospectively processed FFPE samples, or alternative sequencing approaches such as targeted panels—limits our ability to identify specific contributions to the observed sequencing failure. The high loss rate observed likely reflects workflow-dependent feasibility, driven by intrinsic DNA degradation associated with FFPE, conservative analytical thresholds, and the broader capture requirements inherent in WES compared with smaller targeted panels.

Considering that low tumor cellularity can compromise the identification of somatic events [36], only samples containing at least 20% tumor cells were included in our study. Also, samples were required to meet a minimum DNA input threshold of 100 ng to proceed with WES library preparation. Despite the 94.5% of samples presenting at least 20% of tumor cellularity, 25.7% did not achieve an adequate DNA mass. In the regression analysis, tumor cellularity was not significantly associated with WES validity, and DNA concentration demonstrated only a modest association with sequencing success. This pattern likely reflects the constrained variability of these parameters due to the predefined inclusion thresholds applied during sample selection. Lower DNA concentration and quality can be factors of failure in NGS, even in samples with higher cellularity (>50%) in different tumor types [37,38]. Several key factors are known to affect DNA quality and quantity from FFPE tissue, including cold ischemia time, fixation duration, formalin concentration, formalin-to-tissue volume ratio, storage conditions, and DNA extraction method [33,39,40]. Inadequate formalin fixation can induce chemical and mechanical DNA damage, leading to poor fragmentation and impairing genomic sequencing [41,42]. The preservation protocols adopted on the FFPE samples analyzed in this study were unavailable, precluding direct evaluation of their individual impact. The absence of fixation-related data represents a major limitation and restricts causal interpretation and generalizability of the pre-analytical associations observed in this real-world cohort.

Sample age emerged as a major determinant of sequencing success. In multivariable analysis, increasing sample age and smaller peak fragment size were independently associated with lower WES success. FFPE samples producing valid sequencing data were significantly younger and/or had a larger peak fragment size than those classified as inconclusive. These findings align with previous evidence indicating that, although tumor DNA can technically be recovered from samples stored for more than 30 years [42], prolonged storage accelerates DNA degradation and increases fragmentation over time [43]. The mean sample age was 27.9 months, and older samples exhibited shorter fragment length (average and peak), a critical factor for sequencing. Even within a relatively short storage duration in our cohort, progressive DNA damage appeared sufficient to compromise WES feasibility, underscoring the central role of DNA integrity—rather than quantity alone—in determining sequencing success.

Even after initial screening to exclude samples with pre-analytical deficiencies, only 55.6% of those subjected to WES yielded valid sequencing data. Failures during library preparation (16.1%) and inconclusive sequencing results (28.3%) likely reflect the interplay of multiple factors, including pre-analytical variables, analytical limitations, and intrinsic characteristics of tumor tissue, and the potential presence of PCR inhibitors or residual formalin-induced crosslinks that can impair amplification efficiency despite apparently adequate DNA mass and fragmentation profiles [41,42]. The performance of archival tissue in NGS analyses is strongly influenced by both the chosen DNA extraction method and library preparation strategy [44,45]. DNA fragmentation, reduced DNA input, and chemical damage collectively reduce library complexity, increase duplication rates, and cause uneven coverage across the exome, ultimately compromising effective sequencing depth and reducing variant detection. Reduced effective coverage also contributes to inconclusive results [46,47]. The interdependence of these factors makes it challenging to determine the precise causes of sequencing failure.

Variability in tissue processing and handling across participating centers may have negatively impacted sequencing performance. Given the retrospective and multicenter design of this study, detailed documentation of key pre-analytical parameters, such as fixative concentration, fixation duration, and storage conditions, was unavailable. This lack of standardized control limits causal interpretation, precluding the identification of which specific steps were primarily responsible for DNA degradation and sequencing failure, and reducing the practical specificity of protocol refinement recommendations. This study is also limited by the use of a single sequencing workflow and by the adoption of a conservative minimum DNA input threshold of 100 ng, based on manufacturer recommendations. Although alternative low-input strategies may recover a subset of samples, tumor tissue scarcity and highly variable DNA quality inherent to routine FFPE specimens constrained extensive protocol optimization. Consequently, the reported failure rates may be workflow-dependent and may not fully reflect the performance achievable with newer, FFPE-optimized approaches. Importantly, these limitations reflect real-world challenges commonly encountered in molecular pathology laboratories, reinforcing the translational relevance of our findings.

Our study provides an estimate of sequencing yield in routine archived tumor samples from the Brazilian public health system under real-world multicenter conditions. The high failure rate observed represents not only a technical limitation, but also a substantial loss of potentially actionable genomic information. While sequencing failure may hypothetically limit downstream genomic analyses, our study did not assess variant-level accuracy, concordance, or the detection of actionable alterations, and therefore no direct conclusions regarding loss of clinically relevant information can be drawn. In clinical practice, failed or inconclusive WES may directly limit access to precision oncology strategies (biomarker-driven therapies, clinical trial eligibility, and prognostic stratification). At least 30% of breast and prostate tumors harbor potentially actionable alterations [48,49,50], the loss of sequencing in approximately 2/3 of the evaluated samples may translate into missed therapeutic opportunities and clinical usefulness of NGS in a substantial proportion of patients [51]. This impact cannot be fully determined based on the present data and should be interpreted with caution. Despite this, the findings highlight the urgent need for standardized protocols that regulate sample collection, fixation, storage, and DNA extraction to optimize sequencing success. Enhanced awareness of sample quality and harmonization of pre- and post-sequencing workflows are crucial for improving the clinical utility of genomic data derived from FFPE samples. Strengthening these processes will facilitate the broader implementation of precision oncology and ultimately contribute to improved patient outcomes.

## 5. Conclusions

This study identified a high failure rate in WES analysis of archival FFPE tumor samples, exceeding rates reported in previous studies, with valid sequencing data obtained in only 36.7% of all assessed specimens. These findings underscore the significant challenges associated with obtaining reliable genomic data from archival, variably processed FFPE material in real-world multicenter settings. Sample age and DNA fragmentation emerged as key determinants of WES success, suggesting that newer samples should be prioritized whenever feasible for molecular testing. Importantly, this study focused on analytical feasibility and sequencing performance metrics rather than variant-level accuracy or clinical validation. Therefore, the findings should not measure the diagnostic reliability or clinical performance of WES in FFPE samples. The lack of detailed fixation-related parameters and the evaluation of a single sequence workflow represent important limitations for a robust interpretation of the results. The adoption of standardized sample handling protocols and assessment of alternative sequencing strategies may enhance the clinical yield of NGS and support the expansion of precision oncology.

## Figures and Tables

**Table 1:** Pre-analytical parameters in breast and prostate cancer samples.

	Total Mean (SD)	*n*	Breast Cancer Mean (SD)	*n*	Prostate Cancer Mean (SD)	*p*-Value
Sample age (months)	27.9 (32.4)	252	20.6 (19.5)	192	37.6 (42.0)	<0.001
Cellularity (%)	59.8 (25.1)	248	49.0 (21.3)	192	73.9 (22.7)	<0.001
Quantification (ng/µL)	15.4 (20.2)	230	18.2 (21.4)	185 *	11.9 (18.2)	<0.001
Average fragment length (bp)	1105.6 (379.0)	187	1100.9 (359.5)	121	1112.9 (408.7)	0.850
Peak fragment size (bp)	658.3 (501.1)	184	632.0 (443.9)	120	698.5 (577.6)	0.802

Data are presented as means and standard deviations and compared using the Mann–Whitney U test. * One outlier removed.

**Table 2:** NGS quality control metrics in breast cancer (*n* = 146) and prostate cancer (*n* = 100) samples.

	Total Mean (SD)	Breast Cancer Mean (SD)	Prostate Cancer Mean (SD)	*p*-Value
Average autosomal coverage of target regions (×)	420.8 (220.8)	431.9 (222.0)	404.6 (219.1)	0.397
Estimated read length (bp)	116.9 (13.5)	117.52 (12.7)	115.9 (14.6)	0.259
Duplicate marked reads (%)	0.4 (0.2)	0.4 (0.2)	0.5 (0.2)	0.601
Target region with 500× coverage (%)	31.4 (27.6)	32.44 (27.0)	29.9 (28.5)	0.310
Target region with 100× coverage (%)	86.8 (22.1)	87.33 (22.3)	85.9 (21.9)	0.929

Data are presented as means and standard deviations and compared using the Mann–Whitney U test.

**Table 3:** Pre-analytical parameters of BC and PC samples according to NGS results.

	Library Failure (*n* = 47)	Inconclusive NGS (*n* = 83)	Valid NGS Data (*n* = 163)	*p*-Value
Sample age (months)	26.4 (24.5)	39.3 (40.7)	16.2 (13.4)	<0.001
Cellularity (%)	64.8 (21.2)	61.3 (21.4)	61.4 (23.0)	0.631
Quantification (ng/µL)	15.4 (16.8)	23.6 (21.7)	21.2 (22.5)	0.013
Average fragment length (bp)	825.3 (241.3)	984.8 (259.0)	1311.2 (331.7)	<0.001
Peak fragment size (bp)	370.2 (231.6)	489.9 (319.3)	879.3 (551.8)	<0.001

Data are presented as means and standard deviations and compared using the Kruskal–Wallis test.

**Table 4:** NGS quality control metrics among BC and PC samples with inconclusive and valid NGS data.

	Inconclusive NGS (*n* = 83)	Valid NGS Data (*n* = 163)	*p*-Value
Average autosomal coverage of target regions (×)	194.1 (120.2)	536.3 (163.3)	<0.001
Estimated read length (bp)	107.7 (13.4)	121.5 (10.9)	<0.001
Duplicate marked reads (%)	0.6 (0.1)	0.4 (0.2)	<0.001
Target region with 500× coverage (%)	5.3 (8.7)	44.7 (24.2)	<0.001
Target region with 100× coverage (%)	65.4 (27.6)	97.6 (1.7)	<0.001

Data are presented as means and standard deviations and compared using the Mann–Whitney U test.

## Data Availability

The original contributions presented in the study are included in the article, further inquiries can be directed to the corresponding author.

## Supplementary Materials

The following supporting information can be downloaded at: https://www.mdpi.com/article/10.3390/diagnostics16111595/s1, Figure S1: Correlations between autosomal coverage and pre-analytical parameters in BC and PC samples. Spearman correlation between average autosomal coverage and tumor cellularity (A), and DNA concentration (B). Each point represents an individual sample (red for BC and blue for PC). The solid line indicates the fitted correlation, with the shaded area representing the confidence interval. Spearman’s correlation coefficient and the corresponding *p*-value are shown for each analysis. Table S1: Multivariable Poisson Regression Analysis of Independent Predictors of WES Success.

## Abbreviations

The following abbreviations are used in this manuscript:

NGS	Next-generation sequencing
WES	Whole-exome sequencing
BC	Breast cancer
PC	Prostate cancer
CGP	Comprehensive genomic profiling
FFPE	Formalin-fixed paraffin-embedded
gDNA	Genomic DNA
SD	Standard deviation
bp	Base pair
