# Supplementary material for: Pre-Analytical and Analytical Challenges in Whole-Exome Sequencing of Formalin-Fixed Paraffin-Embedded Breast and Prostate Cancer Tissue: A Real-World Multicenter Study
Source: Diagnostics (Basel). 2026 May 23;16(11):1595. doi: 10.3390/diagnostics16111595 (PMC13256900; doi:10.3390/diagnostics16111595)
Supplement: Supplementary file 1 [file diagnostics-16-01595-s001.zip › diagnostics-4130511-supplementary.pdf]

SUPPLEMENTARY MATERIALS

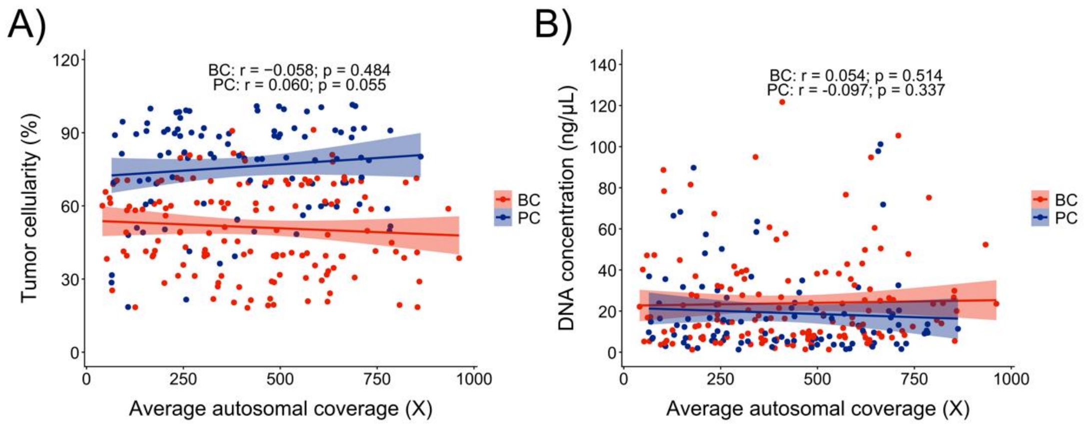

Figure S1. Correlations between autosomal coverage and pre-analytical parameters in BC and PC samples. Spearman correlation between average autosomal coverage and tumor cellularity (A), and DNA concentration (B). Each point represents an individual sample (red for BC and blue for PC). The solid line indicates the fitted correlation, with the shaded area representing the confidence interval. Spearman's correlation coefficient and the corresponding p-value are shown for each analysis.

Table S1. Multivariable Poisson Regression Analysis of Independent Predictors of WES Success

Variable	Univariate PR (95% CI)	Adjusted PR (95% CI)
Sample age (months)	0.80 (0.74-0.87)	0.84 (0.78-0.91)
Cellularity (%)	1.00 (0.94-1.06)	0.98 (0.93-1.04)
Quantification (ng/µL)	0.95 (0.86-1.04)	0.92 (0.85-1.00)
Peak fragment size (bp)	1.66 (1.46-1.90)	1.51 (1.31-1.74)

Prevalence ratios (PR) and 95% confidence intervals (CI) were estimated using Poisson regression models with robust variance. Variables were entered individually in univariate models and simultaneously in the multivariable model.
